# Oncolytic Adenoviral Vector-Mediated Expression of an Anti-PD-L1-scFv Improves Anti-Tumoral Efficacy in a Melanoma Mouse Model

**DOI:** 10.3389/fonc.2022.902190

**Published:** 2022-05-20

**Authors:** Maria Vitale, Filippo Scialò, Margherita Passariello, Eleonora Leggiero, Anna D’Agostino, Lorella Tripodi, Laura Gentile, Andrea Bianco, Giuseppe Castaldo, Vincenzo Cerullo, Claudia De Lorenzo, Lucio Pastore

**Affiliations:** ^1^ Dipartimento di Medicina Molecolare e Biotecnologie Mediche, Università di Napoli Federico II, Naples, Italy; ^2^ CEINGE-Biotecnologie Avanzate, Naples, Italy; ^3^ Dipartimento di Scienze Mediche Traslazionali, Università della Campania “L. Vanvitelli”, Naples, Italy; ^4^ Laboratory of Immunovirotherapy, Drug Research Program, Faculty of Pharmacy, University of Helsinki, Helsinki, Finland

**Keywords:** oncolytic virotherapy, oncolytic adenoviruses, programmed death ligand 1 (PD-L1), Programmed cell death 1 (PD-1), single-chain variable antibody fragment (scFv), B16.OVA cells, C57BL/6J mice

## Abstract

Oncolytic virotherapy is an emerging therapeutic approach based on replication-competent viruses able to selectively infect and destroy cancer cells, inducing the release of tumor-associated antigens and thereby recruiting immune cells with a subsequent increase in antitumoral immune response. To increase the anticancer activity, we engineered a specific oncolytic adenovirus expressing a single-chain variable fragment of an antibody against PD-L1 to combine blockage of PD-1/PD-L1 interaction with the antitumoral activity of Onc.Ad5. To assess its efficacy, we infected B16.OVA cells, a murine model of melanoma, with Ad5Δ24 -anti-PD-L1-scFv and then co-cultured them with C57BL/6J naïve splenocytes. We observed that the combinatorial treatments were significantly more effective in inducing cancer cell death. Furthermore, we assessed the efficacy of intratumoral administrations of Ad5Δ24-anti-PD-L1-scFv in C57BL/6J mice engrafted with B16.OVA and compared this treatment to that of the parental Ad5Δ24 or placebo. Treatment with the scFv-expressing Onc.Ad induced a marked reduction of tumor growth concerning the parental Onc.Ad. Additionally, the evaluation of the lymphocytic population infiltrating the treated tumor reveals a favorable immune profile with an enhancement of the CD8^+^ population. These data suggest that Onc.Ad-mediated expression of immune checkpoint inhibitors increases oncolytic virotherapy efficacy and could be an effective and promising tool for cancer treatments, opening a new way into cancer therapy.

## Introduction

**Figure 1:**
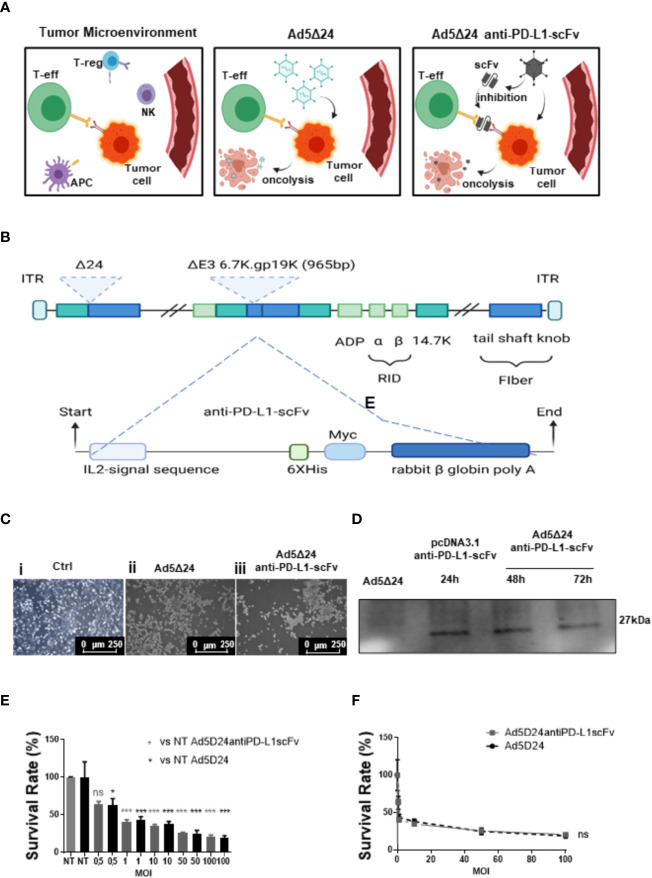
Design and characterization of a novel Ad5delta24-anti-PD-L1-scFv. **(A)** The interaction between PD-L1 on the surface of tumor cells and PD-1 on the surface of T cells induces as a result the immune escape (left panel). The injection of Ad5Δ24 into TME, results in a potent lytic effect on tumor cells, leading to a tumor-specific T cells response (central panel). The injection of Ad5Δ24-anti-PD-L1-scFv into TME, induces not only a potent lytic effect on tumor cells, due to Ov. Ads, but also, inhibit the interaction between PD-L1 and PD-1 through the expression of an scFv, resulting in a failure of immune escape (right panel). Regulatory T cells (Tregs), natural killer (NK) cells, tumor cells, and effector T (Teff) cells in the tumor microenvironment (TME). **(B)** A schematic representation of oncolytic adenovirus serotype 5 (Ad5) DNA viral backbone containing a deletion of 24 bp (Δ24 or D24) in the Rentinoblastoma (Rb) binding constant region 2 of E1 gene. Dashed line indicate the insertion site of a nucleic acid sequence encoding a single chain fragment variable (scFv) anti-PD-L1, in the place of the deleted gp 19k/6 in the adenoviral E3 region. **(C)** A549 cells were infected with 50MOI of Ad5Δ24 (ii) or Ad5Δ24-anti-PD-L1-scFv (iii). Images show that compared to control cells (i) at 48hrs post-infection a potent lytic effect was noted confirming that the remodeling of Ad5delta24 DNA does not interfere with its oncolytic action. **(D)** To test the scFv production A549 cells were infected with 500 MOI of Ad5Δ24-anti-PD-L1-scFv, and the media was collected at 48 and 72 h to test the presence of scFv by using an anti-Hys-tag as primary antibody. Collected media from A549 cells infected with Ad5Δ24 were used as a negative control, while media from A549 cells transfected with pcDNA 3.1+ scFv anti-PD-L1 at 24 h was used as positive control. A band of 27 kD was detected in the media of infected or transfected cells while was absent in the negative control. **(E)** A549 were infected with increasing MOI of Ad5Δ24 (in black) or Ad5delta24-anti-PD-L1-scFv (in gray) and cell count was performed at 48hrs post-infection. The graphs show the survival rate expressed in the percentage of the cell still alive and compared to the uninfected control. The data were analyzed with GraphPad Prism version 5.02 through One-way analysis of variance. The significance was evaluated with Turkey’s Multiple Comparison Test comparing each condition to the uninfected control. In the graph, SEM is reported for each column. **(F)** Comparison of survival rate in A549 infected with different MOI of Ad5Δ24 or Ad5delta24-anti-PD-L1-scFv at 48 h post-infection. The graphics were obtained through GraphPad Prism 5.02 version. *≤ 0.05, *** ≤ 0.001 and ns, not significant.

Despite scientific efforts and the development of new therapies, cancer remains one of the leading causes of death in the 21st century (1). Recently, some promising findings involving the use of the immune system (IS) as a weapon against tumors have been reported (2). Indeed, the IS can be activated, making it able to identify and eradicate tumor cells (3). However, some hurdles make the work difficult for the IS; the most relevant of these is the tumor microenvironment (TME). The TME is a very complex structure with multiple components (4), which altogether create an immune-suppressive environment and induce the immune-escape of cancer cells (**Figure 1A**) (5). Programmed cell death ligand 1 (PD-L1)/programmed cell death (PD-1) interaction and its downstream pathway plays a crucial role in cancer cell immune-escape (6). At TME, the interaction between PD-L1 on the surface of tumor cells and PD-1 on the surface of T cells induces suppression of T-cell function, causing T-cell tolerance, inhibition of their proliferation, and lowering their cytokine production. This combination produces, as a result, the immune escape of tumor cells (7, 8). To prevent PD-1/PD-L1 interaction several approaches have been developed; the most popular one consists of the use of antibodies (Abs) against one of the two partners, thus interfering with their binding (9, 10). Clinical efficacy and safety of monoclonal Abs (mAbs) have been demonstrated in several studies (11–14), and recently, different types of antibody-like proteins have been developed: they are capable of antigen-binding but have modifications that change some of their properties. The single-chain Fragment variable (scFv) is an antibody fragment made up of the variable regions of heavy (VH) and light chains (VL) joined by a flexible linker peptide, and it is the smallest immunoglobulin fragment endowed with antigen-binding activities. The scFv smaller size, compared to that of the whole mAbs, could offer several advantages in therapeutic applications, such as: i) major penetration capability into the tissue and, in particular, into the tumor; ii) Efficient localization at the tumor sites and no up-take by the kidney; iii) faster blood clearance than the whole-sized antibody; and iv) adaptable size for development of specific viral and non-viral targeting vectors for therapeutic gene delivery. Additionally, to overcome this hostile and immunosuppressive TME, strategies that involve the use of oncolytic viruses (OVs) have been adopted (15). OVs can exert anticancer activity in different ways: i) virus-mediated direct lysis of cancer cells mediates the release of tumor neoantigens (TNAs) and tumor-associated antigens (TAAs) that induce a tumor-specific T-cell response; ii) infected cells can promote a potent inflammatory response by stimulating cytokine production, leading to a lower immunosuppressive TME; and iii) induction of immunogenic cell death (ICD) due to the release of danger-associated molecular patterns (DAMPs) and pathogen-associated molecular patterns (PAMPs) as viral DNA or capsid proteins (16, 17). Adenoviruses (Ads) are among the most extensively studied OVs because their genome can be easily modified (18) without interfering with their capacity to infect host cells (19). After two decades of clinical studies, Ads appear safe and can be used as effective therapies against cancer (20). They can be engineered to express one or multiple transgenes (20) and can accommodate a thousand base pairs of extra genomic DNA. In this study, we decided to evaluate the efficacy of an engineered Ad5Δ24 expressing an anti-PD-L1-scFv (Ad5Δ24-anti-PD-L1-scFv). This approach can exert antitumoral activity in two ways: i) reduction of immune escape following the mobilization of the immune system, principally CD8^+^ T cells; and ii) Ad-mediated cell lysis with consequent TANs and TAAs release (**Figure 1A**). In other words, the immune system can be awakened against tumor cells killing them with mechanisms that are properly used for canonical defense (21).

## Materials and Methods

### Cloning Techniques

The expression cassette for the anti-PD-L1scFv has been excised by pcDNA3.1-anti-PD-L1 (Proteogenix) with BsiWI and MfeI (New England Biolabs) digestion and inserted into pTHSN, generating pTHSN-anti-PD-L1. The resulting shuttle vector with the gene of interest (pTHSN-scFv-anti-PD-L1 plasmid) was recombined with pAd5Δ24 (IVT lab, Faculty of Pharmacy, Helsinki) in the *Escherichia coli* BJ5183 strain (Agilent) *via* electroporation. The electroporation was performed using cuvettes according to the standard protocol from Bi-orad and bacterial cells were plated on LB-agar with kanamycin resistance.

### ELISA

To confirm the binding specificity of the purified immunomodulatory scFv, ELISA assays were performed on both human and mouse chimeric proteins (coated at 5 μg/ml on microplates), and untreated or activated hPBMCs. The ELISA assays on coated chimeric protein were performed by coating NuncTM flat-bottom 96-well plates (ThermoFisher Scientific) with 5 μg/ml of recombinant proteins in a solution of 0.05 M NaHCO3 for 72 h at 37°C. After blocking off the coated 96-well plates with 5% nonfat dry milk in PBS for 1 h at 37°C, the purified scFv was added at increasing concentrations (10–200 nM) to the plates in 2.5% nonfat dry milk in PBS and incubated for 2 h at room temperature by gently shaking. Cell ELISA assays were performed by plating the cells in round-bottom 96-well plates (2 × 10E5 lymphocytes for each well) and incubating them with increasing concentrations of the scFv in 2.5% nonfat dry milk for 2 h at room temperature with gentle agitation. After the incubation with the primary antibodies, extensive washes were carried out with PBS, then the plates were incubated with an appropriate HRP-conjugated antibody for 1 h at room temperature, washed again, and incubated with 3,3’,5,5’-tetramethylbenzidine (Sigma-Aldrich) reagent for 10 min before quenching with an equal volume of 1 N HCl. Absorbance at 450 nm was measured by the Envision plate reader (Perkin Elmer, 2102).

### Competitive ELISA Assays

To investigate the ability of the selected anti-PD-L1-scFv to compete in the PD-L1/PD-1 or PD-L1/B7.1 binding, competitive ELISA assays were performed by testing the binding of each biotinylated chimeric protein (PD-1/Fc or B7.1/Fc) to PD-L1 in the absence or presence of unlabeled competitive scFv. For this aim, a 96-well plate was coated with 200 ng/ml of PD-L1 recombinant protein in 0.005 M NaHCO3 solution for 72 h at 4°C. Then, the PD-L1 coated plate was pre-incubated with competitor scFv (at a 10:1 M/M excess ratio), and then further treated with biotinylated PD-1 or B7.1 chimeric proteins, which were added to the plate at the same concentrations of competitive antibodies (2 μg/ml). For detecting bound biotinylated proteins, HRP-conjugated Streptavidin (Biorad) was added to the plate, whereas an anti-human antibody was used in parallel assays for the detection of bound anti-PD-L1 antibodies. The error bars were based on the results obtained in triplicate by at least two independent experiments.

### Adenovirus Production and Purification

The replication-competent pAd5Δ24 adenovirus was provided by the IVT lab (IVT lab, Faculty of Pharmacy, Helsinki). The plasmid containing the anti-PD-L1-scFv gene was provided by Proteogenix. A549 cells were transfected with Lipofectamine 2000 (Invitrogen) according to the instructions of the manufacturer. After 14 days, cells were harvested, centrifuged at 1,000 rpm for 10 min, and stored at −80°C. Then, we performed three freeze-thaw cycles to lyse cells and obtain virions. The supernatant was collected, treated with DNase, and then subjected to two rounds of ultracentrifugation in a CsCl (Roche) gradient (2.5 ml of CsCl 1.45 gr/ml and 3 ml of CsCl 1.25 gr/ml) for 2 h and, subsequently, for 20 h at 27,000 rpm at 4°C. The viral band was collected and transferred to a dialysis cassette (Thermo Scientific) with 1 L of dialyzing solution (TM: 10 mM Tris–HCl, pH 8.0; 2 mM MgCl2). After 2 h, the TM solution was substituted with 1 L of freezing solution (10 mM Tris–HCl, pH 8.0; 2 mM MgCl2; 4% sucrose) and dialyzed overnight. Finally, the virus was collected, aliquoted, and stored at −80°C. The concentration was measured as the number of viral particles, determining absorbance at 260 nm. Furthermore, virus infectious units (ifu) have been calculated using the Adeno-XTM Rapid Titer Kit (Clontech Laboratories Inc.) based on immunodetection of the adenoviral hexon protein in transduced cells, according to the instructions of the manufacturer. The titer of Ad5Δ24-anti-PD-L1-scFv after amplification and purification was 2.4 × 10^12^ vp/ml. The titer of Ad5Δ24, was 2.2 × 10^13^ vp/ml.

### PCR Analysis

Viral DNA was extracted with a standard phenol/chloroform (Sigma) extraction protocol. DNA is quantified with Nanodrop (Euroclone) and analyzed by PCR. We used HotMasterMix from Quantabio, following the instructions of the manufacturer. Primers were synthesized by the DNA LAB facility at the CEINGE-Biotecnologie Avanzate:

Forward oligonucleotide -5’AAAACACCACCCTCCTTACCT3’-

Reverse oligonucleotide -3’GCTCCGTTCAAATCCTCTTCG5’ -.

Their complementary regions are at both ends of the transgene.

### Cell Culture and Transfection

The A549 and SK-MEL-28 cells (provided by CEINGE-Biotecnologie Avanzate cell culture facility) were cultured respectively with alpha-MEM and D-MEM (Sigma Aldrich), supplemented with 10% fetal bovine serum (FBS, Gibco), 50 U penicillin/50 μg streptomycin (Microgem) and 4 mM L-glutamine (Gibco) in a humified incubator at 37°C with 5% CO2. A549 were seeded in a 60-mm dish and transfected the following day with Lipofectamine 2000 transfection reagent (Life Technologies, USA), according to the instructions of the manufacturer. B16.OVA cells (donated by the IVT lab of Vincenzo Cerullo, Helsinki, Finland) were cultured with RPMI (Gibco) supplemented with 10% FBS, 50 U penicillin/50 μg streptomycin, and 4 mM L-glutamine in a humified incubator at 37°C with 5% CO2.

### Co-Culture Experiments

B16-OVA cell lines were seeded in 96-well plates and were infected with a range of 1 to 100 MOI of modified or un-modified Ads as a control. The splenocytes were extracted from the C57BL/6J naïve spleen, smashed with a cell strainer 70 μm Nylon (Falcon), and cultured in R10 medium (RPMI supplemented with 10% FBS, 50 U penicillin/50 μg streptomycin, and 4 mM L-glutamine, 1% sodium pyruvate 100 m, and 0.1% 2beta-mercaptoethanol 50 mM Gibco). The splenocytes were primed for 24 h with 100 MOI of Ad5Δ24 or Ad5Δ24-anti-PD-L1-scFv in a humified incubator at 37°C with 5% CO2. After 24 h, primed splenocytes were added to the B16-OVA cells. After 24 h B16-OVA cells that were still alive were counted with the trypan blue (0.4%, Sigma) method.

### Western Blot Analysis

Three 100 mm dishes of A549 were infected with 500 MOI of our modified Ads; infections were collected at three different times (24, 48, and 72 h), centrifuged at 1,000 rpm for 5 min, and subjected to 4 freeze-thaw cycles. Later, 40 µl of supernatant of each sample was added with 5 µl of loading + reducing buffer (Life Technologies) and denatured at 75°C for 5 min. These samples were loaded onto a 12% SDS-polyacrylamide gel and separated for ~3 h at 80 V. Proteins were electrophoretically transferred onto a nitrocellulose membrane (Life Technologies) and then blocked with 5% BSA in PBS/Tween (0.1%) for 1 h at 37°C to prevent non-specific antibody binding. Subsequent immunostaining was obtained using an anti-His HPR-conjugated antibody that recognizes His-tag in the an-ti-PD-L1-scFv. It was diluted 1:2,500 in 1% BSA with PBS/Tween (0.1%). The Pierce^®^ ECL Western Blotting Substrate from Thermo Scientific was used according to the instructions of the manufacturer to reveal the signal. We cannot normalize the sample using a housekeeping protein but for volume used (22).

### Animal Studies

Animal studies were conducted by the National Institutes of Health guidelines in accordance with ethical and safety rules and guidelines for the use of animal studies in biomedical research provided by relevant Italian laws and European Union’s directives (no.86/609/EC). The Ministry of Health has approved this work. All efforts were made to minimize the suffering of the animal. Food and water were provided *ad libitum*. For all the experiments, we used an 8-week-old female C57BL/6J (Jackson Laboratory) engrafted subcutaneously into the right and left flank with 3 × 105 B16.OVA cells. The viral dose was 2 × 1,010 vp/kg and was injected directly into the tumor.

### Flow Cytometry

Surface staining was conducted using the following antibodies: CD3 PerCP-Cy5.5 (eBio-science, San Diego, California); CD8 FITC (eBioscience); CD4 PeCy7 (eBioscience); CD45R/B220 APC (Biolegend, San Diego, California); anti-mouse CD45 APC-Cy7 (Sony Biotechnology, San Jose, California). Cells were initially stained with surface markers (CD3, CD8, CD4, CD45R/B220, and CD45) and then stained for FOXP3 (Fox-P3/Transcription Factor Staining Buffer Set, eBioscience) using a protocol for nuclear detection, according to the instructions of the manufacturer.

### Statistical Analysis

Statistical analysis was performed using GraphPad Prism 5.02 for Windows (GraphPad Software, La Jolla, California, USA) as reported in each figure legend. As reported in the figure legend, the statistical significance was evaluated with a one-way analysis of variance (ANOVA) analysis with a p-value <0.05. Tukey’s Multiple Comparison test was used to compare the difference between un-treated values and other values. All data are reported as ± SEM.

## Results

### Ad5Δ24-Anti-PD-L1-scFv Infection Induces Tumor Cell Lysis and Interferes in PD-L1/PD-1 Binding

We isolated a novel human anti-PDL1-scFv by phage display technology that allows for selecting binders from large antibody phage libraries containing up to 10^10^ different variants. The isolation of anti-PD-L1 scFvs consisted of alternate panning rounds of the phage antibody library on live activated hPBMCs expressing the target protein and on immobilized recombinant purified targets to increase the specificity. This approach guaranteed the efficient selection of a large number of clones with high specificity for PD-L1 antigen in its native conformation, like that presented on the cell membrane. Subtractive selection rounds to subtract the phages that recognize the Fc domain present in the PD-L1/Fc chimeric proteins used in the following two parallel rounds, as previously reported for other selections (23–25) The screening of scFvs cross-reactive for human and mouse PD-L1 proteins was performed by parallel ELISA assays on both human and mouse recombinant proteins. We identified a clone capable of recognizing both mouse and human targets and we expressed it in bacteria for its biological characterization. After purification from the periplasmic extract by IMAC (**Supplementary Figures 1A, B**), we tested it by ELISA assays for its binding to recombinant PD-L1 at increasing concentrations (**Supplementary Figure 1C**). The scFv showed high affinity for its target with an obvious Kd value of 4nM. Then we tested for its ability to inhibit the interaction between PD-L1/PD-1 and PD-L1/B7.1, the principal receptors involved in the interaction with PD-L1. To this aim, we performed a competitive ELISA by measuring the binding to immobilized PD-L1 of biotinylated PD-1 or B7.1 in the absence or presence of saturating concentrations of unlabeled anti-PD-L1-scFv. As shown in **Supplementary Figure 1D**, the samples treated with anti-PD-L1-scFv show a reduction in the absorbance compared to the untreated controls in both cases. These data suggest that the selected anti-PD-L1-scFv can interfere with the interaction of the ligand with its receptors, demonstrating a significant ability to bind to PD-L1 and, consequently, inhibit the interaction with PD-1 and B7. Afterward, we engineered Ad5Δ24 introducing an expression cassette containing a cDNA encoding the anti-PD-L1-scFv (Ad5Δ24-anti-PD-L1-scFv, **Figure 1B**) for subsequent *in vitro* and *in vivo* evaluation. Expression and secretion of the anti-PD-L1-scFv were confirmed by infecting adenocarcinoma human alveolar basal epithelial cells (A549) with a dose of 50 vp/cell of either modified or un-modified Ad, used as a control, and assessing scFv secretion by Western blotting analysis with an anti-Hys-tag mAb on media conditioned with infected cells for 48 and 72 h. Cells infected with Ad5Δ24-anti-PD-L1-scFv showed a cytopathic effect 24 h post-infection and at 48 h, cells began to die (**Figure 1C**). The expression and secretion of the anti-PD-L1-scFv were evaluated until 72 h post-infection. As shown in **Figure 1D**, expression and secretion of scFv in the media are maintained for up to 72 h, suggesting that Ad5Δ24-anti-PD-L1-scFv does not only replicate, inducing cytopathic and lytic effects, but also expresses and secretes scFv encoded by the expression cassette, which is stable in the serum at 37°C. Furthermore, we evaluated the effect of Ad5Δ24-anti-PD-L1-scFv compared to Ad5Δ24 on A549, a PD-L1^−^ cell line. Therefore, we infected A549 with different multiplicity of infection (MOI) of either Ad5Δ24-anti-PD-L1-scFv or Ad5Δ24, used as a control, starting from an MOI of 0.5 up to 100. Both Ads induce the same effect on this cell line, with a mortality rate of almost 37.5% at 0.5 MOI that increases at high MOI (almost 80% of mortality at 100 MOI) (**Figure 1E**). Additionally, the comparison of the survival rate between the two Ads, related to the increased MOI of the Ads, reveals that both viruses had the same effect on the A549, with differences not significant as shown in **Figure 1F**.

### Ad5Δ24-Anti-PD-L1-scFv Infects and Kills Human Melanoma Cell SK-MEL 28

**Figure 2:**
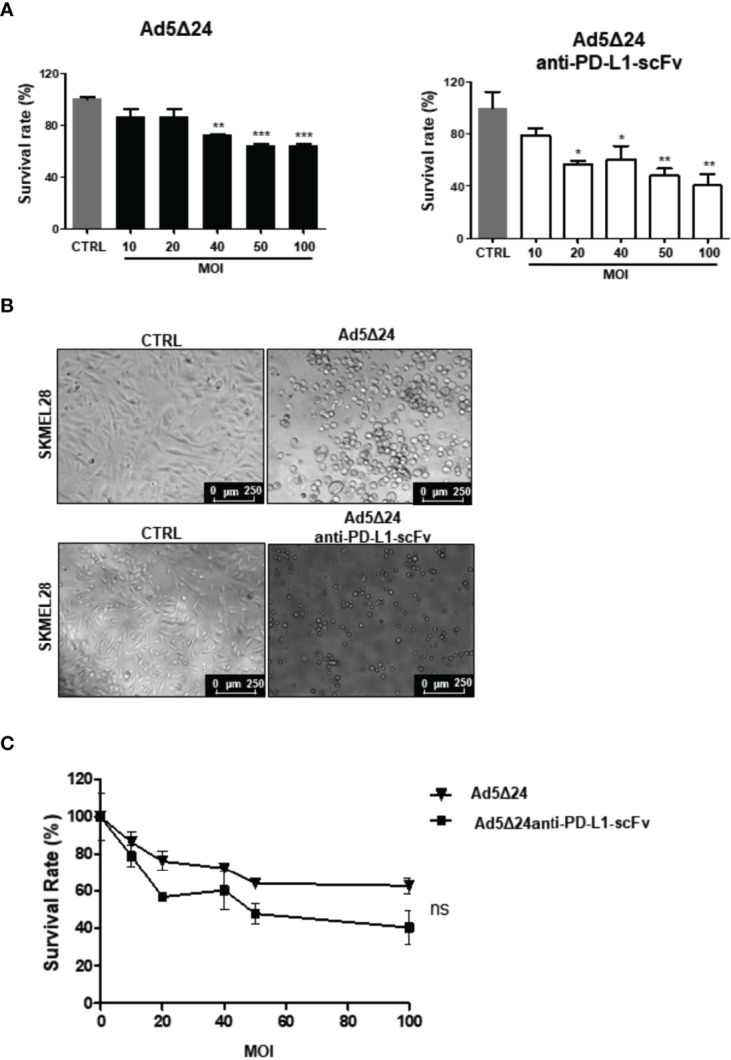
Effects of infection with Ad5Δ24-anti-PD-L1-scFv on human melanoma cell lines SK-MEL28. **(A)** Human melanoma cells line SK-MEL28 were infected with increasing MOI of Ad5Δ24 (in black) or Ad5delta24-anti-PD-L1-scFv (in white) and cell count was performed at 48 h post-infection. The graphs show the survival rate expressed in the percentage of the cell still alive and compared to the uninfected control. The data were analyzed with GraphPad Prism version 5.02 through One-way analysis of variance with a P-value of <0.0001 for Ad5Δ24 and 0.0026 for Ad5delta24-anti-PD-L1-scFv. The significance was evaluated with Turkey’s Multiple Comparison Test comparing each condition to the un-infected control. In the graph, SEM is reported for each column. **(B)** Representative images of SK-MEL28 infected with Ad5Δ24 or Ad5delta24-anti-PD-L1-scFv at 48 h post-infection. The images clearly show the cytopathic effect associated with both Ads while absent in the un-infected control. **(C)** Comparison of survival rate in SK-MEL28 infected with different MOI of Ad5Δ24 or Ad5delta24-anti-PD-L1-scFv at 48 h post-infection. The graphics were obtained through GraphPad Prism 5.02 version. The data were analyzed with GraphPad Prism 5.02 version *via* T-test with a P-value of 0.1797 indicating that the differences were not significant (ns). *≤ 0.05, ** ≤ 0.01 and ***≤ 0.001.

After assessing the ability of Ad5Δ24-anti-PD-L1-scFv to express and secrete the anti-PD-L1-scFv, we evaluated its efficacy *in vitro*. For this aim, we evaluated the effects of Ad5Δ24-anti-PD-L1-scFv on SK MEL 28, a human malignant melanoma cell line. SK-MEL 28 expresses a high level of PD-L1, making this a suitable model to test the engineered Ad (26). Therefore, we infected SK-MEL 28 with a different multiplicity of infection (MOI) of either Ad5Δ24-anti-PD-L1-scFv or Ad5Δ24, used as a control, starting from an MOI of 10 up to 100. At the highest MOI of Ad5Δ24-anti-PD-L1-scFv, only 40% of cells survived, whereas 60% cell survival was observed with Ad5Δ24 (**Figure 2A**). Ad5Δ24-anti-PD-L1-scFv retains the typical cytopathic effect of Ad5Δ24 (**Figure 2B**). Furthermore, at 48 h post-infection, when the replicative cycle of Ad is almost complete, cell counts demonstrated that cell death increased with the higher MOI of the virus, indicating a dose–response correlation as reported in **Figure 2C**. This suggests that expression of the anti-PD-L1-scFv in the absence of the immune system had the same effect as Ad5Δ24, and indeed, the difference between both Ads was not significant (**Figure 2C**).

### Co-Culture of B16.OVA With Naïve C57BL/6J Splenocytes Increases the Anti-Tumor Effects of Ad5Δ24-Anti-PD-L1-scFv

**Figure 3:**
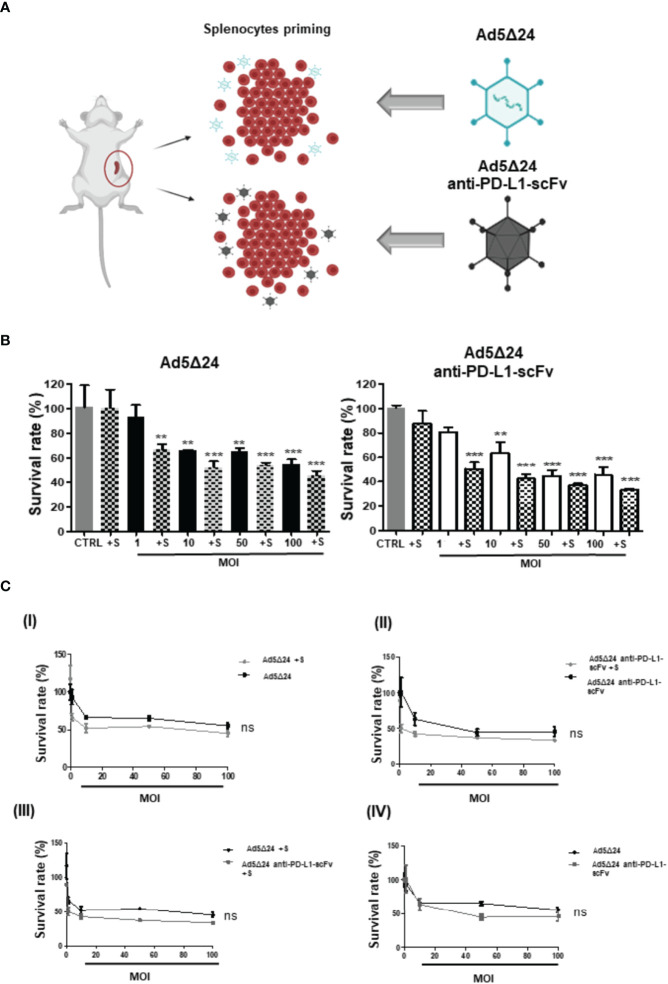
*In vitro* evaluation of Ad5Δ24-anti-PD-L1-scFv infection. **(A)** Schematic representation of splenocytes derived from C57BL/6J (6–8 weeks old) naïve, primed with 50 MOI of Ad5Δ24-anti-PD-L1-scFv or Ad5Δ24 for 16–18 h before the incubation with infected B16 OVA cells. The number of splenocytes was five times more than B16 OVA cells and remains in contact with the cells for 24 (h) **(B)** Mouse melanoma cell line B16. OVA cells were infected with increasing MOI of Ad5Δ24 (in black) or Ad5delta24-anti-PD-L1-scFv (in white). At 24 h post-infection the same number of splenocytes were added to the cells and indicated in the graph with “+s”. Cell viability was assessed at 48 h post-infection. The graphs show the survival rate expressed in the percentage of cells still alive with or without splenocytes and compared to the uninfected control. The data were analyzed with GraphPad Prism version 5.02 through One-way analysis of variance with a p-value of <0.0001 for both Ads. The significance was evaluated with Turkey’s Multiple Comparison Test comparing each condition to the un-infected control. In the graph, SEM is reported for each column. **(C)** I) Comparison of survival rate in B16.OVA infected with different MOI of Ad5Δ24 with and without splenocytes at 48 h post-infection. II) Comparison of survival rate in B16.OVA infected with different MOI of Ad5Δ24-anti-PD-L1-scFv with splenocytes at 48 h post-infection. III) Comparison of survival rate in B16.OVA infected with different MOI of Ad5Δ24 or Ad5Δ24-anti-PD-L1-scFv with splenocytes at 48 h post-infection. IV) Comparison of survival rate in B16.OVA infected with different MOI of Ad5Δ24 or Ad5Δ24-anti-PD-L1-scFv at 48 h post-infection. The dose–response analysis was performed with GraphPad Prism. The statistical analysis was performed GraphPad Prism 5.02 version *via* T-test with respectively p-values of 0.4206, 0.1508, 0.1580, and 0.8413. ** ≤ 0.01 and *** ≤ 0.001 and ns, not significant.

After assessing the ability of Ad5Δ24-anti-PD-L1-scFv to infect and replicate in SK-MEL 28, we determined its ability to infect B16.OVA, a murine melanoma cell line. As reported in the literature, B16.OVA cells express PD-L1 on their surface at constant levels *in vitro* (27), making these cells a suitable model for testing Ad5Δ24-anti-PD-L1-scFv. Furthermore, to mimic the interaction of IS with tumor cells *in vitro*, we used naïve C57BL/6J splenocytes, which contains a heterogeneous immune cell population composed mainly of B and T lymphocytes. We assume that splenocytes could represent a simplified *in vitro* IS, that could enhance the oncolytic effect of the Ads in killing cancerous cells. Therefore, to corroborate our hypothesis, we evaluated tumor cell survival after treatment with Ad5Δ24-anti-PD-L1-scFv or Ad5Δ24 alone or along with splenocytes primed by incubation for 24 h with 100 vp/cell of Ad5Δ24-anti-PD-L1-scFv or Ad5Δ24, respectively (**Figure 3A**). We then infected B16.OVA cells with either Onc.Ads (using MOI from 1 to 100), alone or along with primed splenocytes. Forty-eight hours post-infection, cell counts demonstrated that combinatorial treatments were more effective than Ads alone (**Figure 3B**). Moreover, a combination of Ad5Δ24-anti-PD-L1-scFv and primed splenocytes had a significant efficacy even at the lowest MOI (1 MOI) with only 55% cell survival compared with the 87% observed in B16.OVA cells were treated with Ad5Δ24-anti-PD-L1-scFv alone (**Figure 3B**). Additionally, we plotted these data in different graphics to compare the effects of both Ads at different MOI with or without splenocytes. Thanks to these comparisons, we are able to observe a trend in the reduction of cell survival when scFv is expressed (**Figure 3C**). In detail, in **Figure 3CI**, we compared the Ad5Δ24 infection with and without splenocytes. Even though the differences between treatments were not significant, we report a trend in which the presence of the splenocytes improves the virus efficacy; this is probably due to the ability of the lymphocytic population to recognize tumor cells after infection. In **Figure 3CII**, we compared Ad5Δ24-anti-PD-L1-scFv infection with and without splenocytes. In this graphic, the differences between treatments were insignificant, but the presence of the splenocytes induced a reduction in survival rate of 66% compared to the treatment with the virus only, in which the reduction of the survival rate was 54%. In **Figure 3CIII**, we compared the Ad5Δ24 and Ad5Δ24-anti-PD-L1-scFv with splenocytes. Even in this graphic, differences were insignificant. However, we observed a reduction in survival rate of 66% for the Ad5Δ24-anti-PD-L1-scFv and splenocytes compared to 54% for the Ad5Δ24 and splenocytes. In **Figure 3CIV**, we compared infections with either Ads, that in the absence of the immune system had the same effects on the cells. Indeed, Ad5Δ24 induced a cell death of 45% while Ad5Δ24-anti-PD-L1-scFv induced a cell death of 55%. In summary, these data suggest that the combinatorial treatment (splenocytes and Onc.Ads) seems to show a trend in which the addition of splenocytes induces a reduction in cancer cell survival compared to any single treatment (splenocytes or Onc.Ads alone). In addition, the best results were obtained with the combination of Ad5Δ24-anti-PD-L1-scFv with splenocytes shown in **Figure 3CII**. However, expression of the anti-PD-L1-scFv results in a strengthening of Onc.Ads therapy because it likely blocks PD-1/PD-L1 interactions, stimulating the IS, and in particular, the T cell population, to recognize and kill cancer cells (**Figure 1A**).

### Intratumoral Administration of Ad5Δ24-Anti-PD-L1-scFv Improves OV Efficacy in Reducing Tumor Growth in a Melanoma Mouse Model

**Figure 4:**
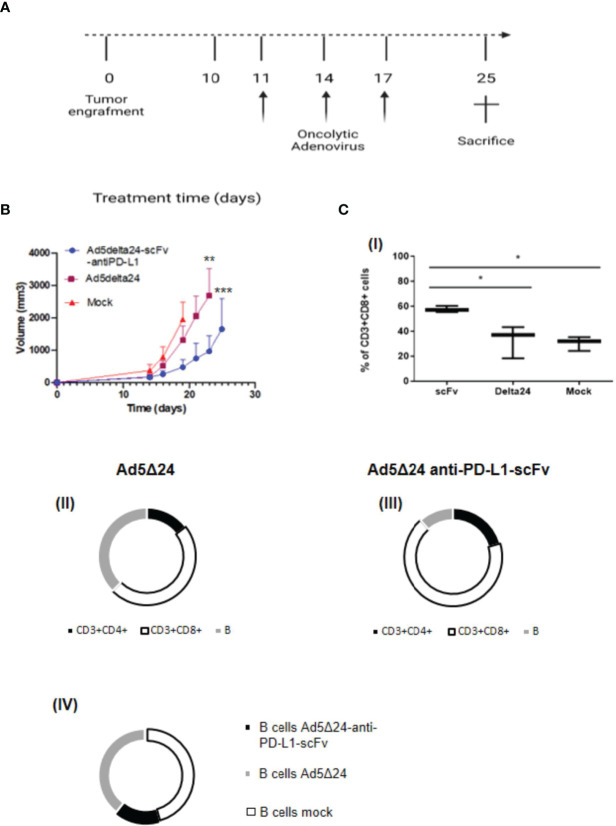
Effect of Ad5Δ24-anti-PD-L1-scFv infection in melanoma mouse model. **(A)** Timeframe of the *in vivo* experiment. Five to six animals per group received subcutaneous B16.OVA melanoma cells (day 0) that were left to grow for 10 days. Then, 2 × 10E10 vp/kg mice of Ad5Δ24 or Ad5Δ24-anti-PD-L1-scFv or PBS were injected intratumorally. The intratumoral injection is repeated at days 11, 14, and 17 while at day 25 mice were sacrificed and tumors were collected. **(B)** Analysis of tumor size described in panel **(A)**. Tumors volumes were analyzed for each experimental group every 2–3 days until the day of sacrifice. The data are plotted as the mean ± SEM. The statistical analysis was performed with GraphPad Prism 5.02 version *via* Spearman test, with a p-value of 0.0028 for Ad5delta24 and 0.0004 for Ad5delta24-scFv-anti-PD-L1. **(C)** Percentage of CD3^+^ CD8^+^ tumor-infiltrating lymphocytes by flow cytometric analysis are plotted by Tukey box and whiskers for each group of animals (I). Statistical analysis was done by two-way ANOVA with a p-value of 0.0134. Flow cytometric analysis of CD3^+^CD4^+^, CD3^+^CD8^+^, and B220^+^ cells in tumor samples from each group of mice (II–III–IV). *≤ 0.05, ** ≤ 0.01 and *** ≤ 0.001.

After assessing the ability of the Ad5Δ24-anti-PD-L1-scFv *in vitro* in SK MEL 28 and B16.OVA, we decided to evaluate its efficacy *in vivo*. For this aim, we implanted 3 × 10^5^ B16.OVA cells into the right and left flanks of 6/7-week-old C57BL/6J female mice. After 10 days, when the tumor size was about 5 mm, we intratumorally administered 2 × 10^10^ vp/kg of either Ad5Δ24-anti-PD-L1-scFv or Ad5Δ24; a control group was treated with PBS. Ads and PBS administrations were repeated three times (11, 14, and 17 days) and tumor growth was evaluated until the day of sacrifice, corresponding to 25 days after B16.OVA cell implantation (**Figure 4A**). Analysis of tumor growth showed that the administration of Ad5Δ24 produced mild growth inhibition, whereas treatment with Ad5Δ24-anti-PD-L1-scFv significantly reduced tumor size at day 19 compared to the other treatments (**Figure 4B**). These data demonstrate that both Onc.Ads are effective in tumor growth inhibition, underlining a stronger effect of Ad5Δ24-anti-PD-L1-scFv. As previously discussed, we expected that scFv expression could contribute to tumor growth inhibition by blocking PD-L1 interaction with PD-1 on effector T cells and, therefore, increasing lymphocyte recruitment. For this aim, we analyzed by flow cytometry the intratumoral CD3^+^CD8^+^ lymphocyte population, namely the tumor-infiltrating lymphocytes (TILs). Among the CD3^+^ positive population, CD8^+^ is increased in tumors treated with Ad5Δ24-anti-PD-L1-scFv compared to the other groups, indicating that the Onc. Ad-induced expression of the anti-PD-L1-scFv potentiates T-cell infiltration in treated tumors (**Figure 4C**). Additionally, the tumor treated with Ad5Δ24 showed a mild increase in CD8^+^, a feature of the Onc. Ads treatment (**Figures 4CI** **)**. Furthermore, immunological analyses performed on TIL (**Table 1**) revealed that administration of Ad5Δ24-anti-PD-L1-scFv induced an expansion of the CD3^+^CD8^+^ cell population; indeed, the percentage is 68% while a milder effect was observed in Ad5Δ24 treated tumors with a percentage of 48% (**Figure 4C II–III**). Finally, the B220^+^ cells were significantly decreased in the group treated with Ad5Δ24-anti-PD-L1-scFv compared to the mock and the group treated with Ad5Δ24 in which the B population was 36 and 37% (**Figure 4CIV**).

**Table 1:** Cytofluorimetric evaluation of TILs.

	B220^+^	CD3^+^CD4^+^	CD3^+^CD8^+^
**Mock**	36%	27%	37%
**Ad5Δ24**	37%	15%	48%
**Ad5Δ24scFv-anti-PD-L1**	11%	21%	68%

We evaluated the mean percentages of TILs population from different tumors treated with PBS (Mock), Ad5Δ24, or Ad5Δ24 anti-PD-L1-scFv. In the groups of animals that received the Ad5Δ24 anti-PD-L1-scFv was observed an enhancement of the CD3^+^CD8^+^ T-cell population. In fact, as can be seen from the table the percentage of CD3^+^CD8^+^ T-cell population in the tumors treated with Ad5Δ24 anti-PD-L1-scFv is 68% compared with 48 and 37% of Ad5Δ24 and Mock, respectively.

## Discussion

Melanoma is the most dangerous and aggressive form of skin cancer. Despite the easier detection and the effectiveness of surgery in the early stages, patients with advanced melanoma have a median survival of 7 months and a 5-year survival rate of less than 10% (13, 26), making melanoma among the more difficult cancers to eradicate. The first therapeutic approaches in melanoma treatment involved the administration of IL-2 and interferon alone or in combination with chemotherapy. Although these treatments have had some success, the most relevant progress has been made with therapies based on the use of ICIs (12). The first ICI therapy for melanoma approved by the FDA was ipilimumab (12, 13). Ipilimumab is a mAb that targets CTLA-4, resulting in a blockade of inhibitory signals allowing T cells to respond against TAAs. An additional extremely relevant target for immunotherapy is the PD-1/PD-L1 binding that also causes a block of T-cell response. Antibodies against PD-1 and PD-L1 block interactions between these proteins (28). Unfortunately, despite several immune-based cancer treatments being currently available, not all patients benefit from them or respond to treatments only for an initial period (13). Additionally, mAb production is expensive and requires maintenance of transfected cells (29), and the Fc domain of mAb induces immunogenicity that can lead to complement fixation or phagocytosis of bound cells interfering with therapeutic effects. Furthermore, systemic administration of mAbs is hampered by several limitations that include: i) unpredictable patient response, ii) low tumor penetration, and iii) slow clearance from the blood with retention in several organs, including the liver (30). Finally, several tumor types have poor lymphocyte infiltration and are defined as “cold” tumors; in these tumors, immunotherapy has a poor effect due to the lack of a suitable number of cells to be primed by TAAs (15, 27). To overcome some of these obstacles, we have decided to develop an anti-PD-L1 antibody in scFv format and express it in an OV. After intratumoral administration, Ad5Δ24 induces an antiviral as well as antitumoral immune response. The antiviral response is directly involved in the initial priming of the antitumoral response, promoting the recruitment of immune cells. It has been observed that OVs can turn a “cold” TME into a “hot” one; therefore, even tumors defined as “desert” for the absence of a lymphocytic population could benefit from a viro-immunotherapeutic treatment (31). Additionally, OVs induce cancer cell lysis, generating the immunogenic cell death signal (ICD) that has a main role in the activation of tumor-specific responses mediated by CD4^+^ helper cells, CD4^+^ and CD8^+^ cells (2). Hence, the association of OVs with classical immunotherapy has been proven extremely powerful (27) in animal models and clinical trials are underway to evaluate this combination in patients. The use of scFvs can also promote a more potent and specific immune response against cancer. Indeed, the smaller size of the scFv overcomes some of the limitations (large size) and possible unwanted side-effects of the whole antibodies, such as non-specific activation of circulating lymphocytes due to prolonged half-life, and promotes more efficient tumor tissue penetration (32). In this work, we have expressed the scFv-anti-PD-L1 in Ad5Δ24. We have chosen the Onc.Ad5Δ24 because of its well-known ability to induce an antitumoral immune response in the immunogenic tumor model of melanoma. Expression of the anti-PD-L1-scFv in Ad5Δ24 is a way to combine the advantages of “passive” immunotherapy with “active” virotherapy, capable of inducing recruitment of immune cells. Recently, it has been demonstrated that OV-based virotherapy is less effective in advanced melanoma management compared to OV virotherapy combined with ICIs, suggesting that the addition of ICIs increases efficacy (33). Furthermore, additional studies have examined the treatment with different OVs in combination or expressing a variety of scFv targeting ICIs (18, 34, 35). For example, Wu et al. engineered a vesicular stomatitis virus (VSV) to express an scFv-anti-PD-L1, demonstrating that this system shows a potent therapeutic effect in a lung carcinoma mouse model (35), while Tanoue et al., in 2017, demonstrated that the PD-L1 mini body expressed by a system of Onc.Ads and helper-dependent Ads (HD-Ad) blocks, with high efficacy, the PD-1/PD-L1 pathway enhanced the antitumoral effect in a prostate cancer engraft mouse model. In this study we explored the combination of the antitumor effect of the Ad5Δ24 Onc.Ad together with the expression of an anti-PD-L1-scFv identified by phage display, in a C57BL/6J melanoma mouse model. The selected anti-PD-L1-scFv binds to PD-L1 thus interfering in PD-1 and B7.1 interactions, representing the principal PD-L1 receptors on immune cells, and does not interfere with the ability of Ad5Δ24 to induce cell death in an *in vitro* model of melanoma, actually enhancing it. Treatment of the B16.OVA murine melanoma cells with different MOI of Ad5Δ24 anti-PD-L1-scFv in the presence of splenocytes, resembling an *in-vitro* simplified tumoral immune system environment, induced a more robust effect compared to the treatments with the virus or splenocytes alone. A major effect on cell death was seen (**Figures 3B, C**), demonstrating that PD-L1-scFv expression enhanced the splenocyte action. Most importantly, *in vivo* intratumoral administration of Ad5Δ24-anti-PD-L1-scFv in murine melanoma model resulted in the prolonged survival of mice compared to the unmodified Ad5Δ24 (**Figure 4B**). Specifically, we observed that untreated mice showed a rapid increase in tumor size, progressive deterioration of physical conditions and, consequently, had to be sacrificed on day 19. In contrast, mice treated with Ad5Δ24 resulted in a mild reduction of tumor progression, highlighting that the sole oncolytic process is not sufficient to completely block tumor growth. In this study, the addition of a control group treated with an anti-PD-L1 mAb was not considered appropriate, since scFvs have different features, as we previously described; in addition, for the purpose of this study, we did not purify the anti-PDL-1 scFv. Finally, we observed that although mice treated with Ad5Δ24-anti-PD-L1-scFv did not show a complete remission, the association of the oncolytic process with PD-L1 inhibition significantly slowed down tumor progression resulting in an important reduction of tumor size and an amelioration of survival condition. In support of the hypothesis that reduction in tumor growth was due to increased antitumoral immune response, we observed an increment in the CD8^+^ T-cell population in the tumors of mice treated with Ad5Δ24 anti-PD-L1-scFv, suggesting that the combination of OV action and PD-L1 inhibition is beneficial for the recruitment and activation of cytotoxic T cells. Furthermore, this data confirmed the main role of the CD8^+^ T-cell population in the detection and destruction of tumor cells (36–38). Additionally, based on the literature and our observation of the T and B population analysis, we assumed that the anti-PD-L1-scFv could also act on Breg, reducing the PD-1 positive Breg population at the tumor site and therefore restoring an anti-tumoral response mediated by the CD8^+^ T-cell population that increased in mice treated with Ad5Δ24-anti-PD-L1-scFv (39). Finally, we plan to compare in future studies the efficacy and toxicity of Ad5Δ24-anti-PD-L1-scFv with the commercially available mAbs and, additionally, to evaluate its efficacy in different tumor models characterized by lower T-cell infiltrations.

## Conclusions

This study demonstrates that combining Onc.Ads with anti-PD-L1-scFv can lead to a more effective antitumoral therapeutic approach by combining active and passive immunotherapy. Although more efforts need to be made to improve the versatility and safety of Onc.Ads, this intriguing approach could have the potential to usher in a new era in cancer treatment.

## Data Availability Statement

The raw data supporting the conclusions of this article will be made available by the authors, without undue reservation.

## Ethics Statement

The animal study was reviewed and approved by the Ministry of Health, Italy.

## Author Contributions

Study concept and design: MV, FS, EL, LT, and LP. Data collection and analysis: AD’A, MV, LG, MP, and LT. Interpretation: MV, FS, MP, EL, VC, CL, and LP. Manuscript preparation: MV, FS, and LP. Critical revision: AB, GC, VC, CL, and LP. All authors listed have made a substantial, direct, and intellectual contribution to the work and approved it for publication.

## Funding

This research was funded by the SATIN CUP B6C1700007-SURF 17061BP000000002 and the University of Naples Federico II, Dipartimento di Medicina Molecolare e Biotecnologie Mediche.

## Conflict of Interest

The authors declare that the research was conducted in the absence of any commercial or financial relationships that could be construed as a potential conflict of interest.

## Publisher’s Note

All claims expressed in this article are solely those of the authors and do not necessarily represent those of their affiliated organizations, or those of the publisher, the editors and the reviewers. Any product that may be evaluated in this article, or claim that may be made by its manufacturer, is not guaranteed or endorsed by the publisher.

## Supplementary Material

Supplementary Figure 1 Figure Legend: Purification and characterization of anti-PD-L1 scFv. **(A)** SDS-PAGE of periplasmic extract of bacteria cells transformed with the cDNA encoding the anti-PD-L1 scFv before (lane 3) and after purification (lanes 4-6) by affinity chromatography. An unrelated scFv (lane 2) was used as a control. **(B)** Western blotting analysis of the eluted fraction (as in lane 5 of panel **A**). **(C)** ELISA assays of the purified scFv on recombinant PD-L1/Fc at increasing concentrations (nM). **(D)** A competitive ELISA assay performed by PD-L1 coating on the plate, followed by saturation with the unlabeled scFv and incubation with biotinylated PD-1 or B7, demonstrate the ability of anti-PD-L1-scFv to interfere in the interaction between PD-1 and B7. Error bars were calculated on the basis of the results obtained in triplicates by at least two independent experiments.
