# Supplementary figures and images for: Oncolytic Adenoviral Vector-Mediated Expression of an Anti-PD-L1-scFv Improves Anti-Tumoral Efficacy in a Melanoma Mouse Model
Source: Front Oncol. 2022 May 20;12:902190. doi: 10.3389/fonc.2022.902190 (PMC9163395; doi:10.3389/fonc.2022.902190)

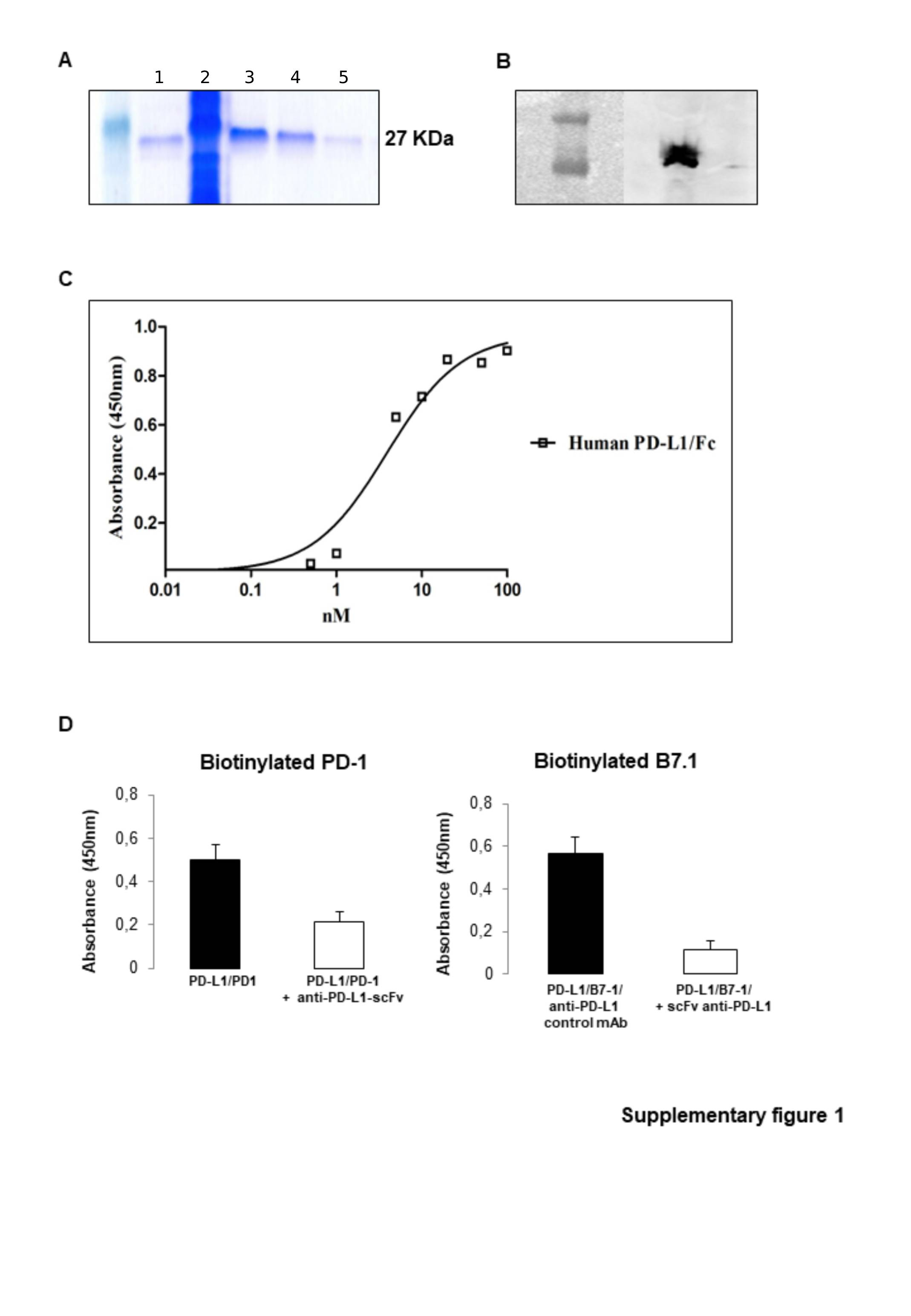

Supplement: Supplementary Figure 1 — Figure Legend: Purification and characterization of anti-PD-L1 scFv. (A) SDS-PAGE of periplasmic extract of bacteria cells transformed with the cDNA encoding the anti-PD-L1 scFv before (lane 3) and after purification (lanes 4-6) by affinity chromatography. An unrelated scFv (lane 2) was used as a control. (B) Western blotting analysis of the eluted fraction (as in lane 5 of panel A). (C) ELISA assays of the purified scFv on recombinant PD-L1/Fc at increasing concentrations (nM). (D) A competitive ELISA assay performed by PD-L1 coating on the plate, followed by saturation with the unlabeled scFv and incubation with biotinylated PD-1 or B7, demonstrate the ability of anti-PD-L1-scFv to interfere in the interaction between PD-1 and B7. Error bars were calculated on the basis of the results obtained in triplicates by at least two independent experiments. [file Image_1.jpeg]
